# Diethyl ether anesthesia inhibits phototropic response in *Arabidopsis thaliana* by disrupting auxin redistribution

**DOI:** 10.1080/15592324.2026.2700912

**Published:** 2026-07-24

**Authors:** Martin Hřivňacký, Marek Rác, Tereza Miksteinová, Christian Luschnig, Andrej Pavlovič

**Affiliations:** a Department of Biophysics, Faculty of Science, Palacký University, Olomouc, Czech Republic; b Laboratory of Growth Regulators, Faculty of Science, Palacký University and Institute of Experimental Botany, Czech Academy of Sciences, Olomouc, Czech Republic; c Institute of Molecular Plant Biology, Department of Biotechnology and Food Science, BOKU University Muthgasse, Wien, Austria

**Keywords:** *Arabidopsis thaliana*, anesthetic, diethyl ether, phototropism, auxin, PHOT1, NPH3, PIN3

## Abstract

Although the term anesthesia is mainly associated with animals, it is well known that anesthetics also affect plants through their actions on membrane properties and protein function. Here, we show that the anesthetic diethyl ether inhibits the phototropic response of *Arabidopsis thaliana* hypocotyls to unidirectional, low-intensity blue light. Diethyl ether acts not only on the inhibition of hypocotyl elongation but also specifically on the inhibition of phototropic signalling. This complex process consists of initial steps, defined by photoactivation of blue-light receptors, phototropins (PHOTs), and further regulators, followed by later steps involving differential accumulation of the plant growth regulator auxin on the organ’s shaded and illuminated sides, leading to heterogeneous cell elongation and bending towards light. Our data reveal that the early steps of phototropic signalling, such as PHOT1 phosphorylation, are insensitive to diethyl ether anesthesia, whereas auxin redistribution was significantly inhibited, as evidenced by the *DR5:GUS* assay and inhibition of auxin transporter PIN3 relocalization. This study therefore provides the first evidence about the inhibitory effect of anesthetics on auxin-mediated growth responses in plants.

## Introduction

The ability of plant organs to adjust growth directionality in response to external light sources is defined as phototropism, and is controlled by phototropins (PHOTs), acting as photoreceptors for blue light (BL)/UVB radiation.^1-3^ In higher plants, two PHOT homologs can be found: PHOT1 and PHOT2,^4^ which, in addition to phototropism, act in further light-induced processes, such as stomatal opening, chloroplast movements, and leaf positioning.^5-10^ In BL-induced phototropism, PHOT1 acts as the primary photoreceptor and functions across a wide range of light intensities, whereas PHOT2 has a predominant role at higher intensities.^8^ ^,^ ^11^ PHOTs are plasma membrane (PM)-associated Ser/Thr protein kinases activated by BL, which is perceived by flavin mononucleotide (FMN) bound within their LOV (Light, Oxygen, or Voltage) domains.^1^ ^,^ ^12^ ^,^ ^13^ Importantly, PHOT1 activation requires autophosphorylation, with a spatial gradient of PHOT1 kinase activity reflecting the irradiance gradient that is considered essential for the initiation of phototropism.^10^ ^,^ ^14-19^ In the early steps of phototropic response, PHOT1 interacts with several proteins, among which the NON-PHOTOTROPIC HYPOCOTYL3 (NPH3) from NPH3/ROOT PHOTOTROPISM2 (RPT2)-like (NRL) family appears essential for phototropic response. This PM-associated protein is essential for the binding of regulatory proteins such as 14-3-3 and acts as a substrate adapter in a CULLIN3-based E3 ubiquitin ligase complex targeting several regulators of phototropism.^8^ ^,^ ^20-24^ BL induces an immediate phosphorylation of a C-terminal 14-3-3 protein binding motif (S744) in NPH3. Subsequent NPH3 association with 14-3-3 proteins is causal for light-induced release of NPH3 from the membrane into the cytosol, together with overall NPH3 dephosphorylation in condensate-like structures. Whilst the mechanistic implications of such NPH3 redistribution are not entirely understood, this process was shown to be essential for lateral redistribution of auxin, triggering differential cell elongation in the course of phototropic organ growth.^25-29^

According to the Cholodny-Went hypothesis, plant tropic responses, including phototropism, are established through the differential accumulation of the plant growth regulator auxin (indole-3-acetic acid (IAA)^8^ ^,^ ^30^). During phototropism, auxin accumulates on the shaded side of the hypocotyl, resulting in greater cell elongation in this area and, consequently, organ bending. Auxin is transported directionally from shoot tips towards the roots through a complex mechanism involving pH-regulated diffusion and polarly localized transporters.^8^ ^,^ ^31-33^ In the apoplast, it naturally occurs in its protonated form, IAAH, which can enter cells either via uptake facilitators or by diffusion through the PM. This is followed by IAA deprotonation in the cytoplasm, allowing for further intercellular IAA transport only by means of specific efflux transporters located at the PM.^34^ In *Arabidopsis thaliana*, members of two protein families have been described to participate in auxin translocation during phototropism: canonical PIN-FORMED efflux carriers (PIN1-4, PIN7) and multi-drug resistance transporters from the ATP-binding cassette B (ABCB) class, especially ABCB19.^21^ ^,^ ^23^ ^,^ ^35-37^ According to previous research, ABCB19 acts as a negative regulator of phototropism and is directly phosphorylated and thus inactivated by PHOT1.^35^ Strikingly, recent research has shown that ABCB19 functions as a brassinosteroid transporter, and so its function in auxin transport associated with phototropism remains unclear.^38^ The role of PINs in directional auxin transport has been analyzed exhaustively, demonstrating that their activity is regulated by expression, phosphorylation, and vesicular trafficking.^36^ ^,^ ^37^ ^,^ ^39-42^ However, in none of these processes has immediate crosstalk between PINs and PHOTs been observed. Phototropic hypocotyl bending, for example, coincides with PIN3 accumulation in endodermal cells on the shaded side in the upper hypocotyl section, thereby facilitating establishment of an auxin gradient.^43^ ^,^ ^44^ This process appears to be regulated by PIN phosphorylation, mediated by PINOID (PID) and D6 PROTEIN KINASE (D6PK) protein kinases as well as by GNOM, an ARF GEF regulator of vesicular trafficking.^43^ ^,^ ^45-50^ Strikingly, whilst reversible PIN phosphorylation and associated modifications in their intracellular sorting appear to be key to mediating phototropic growth responses, a direct role of PHOTs in mediating PIN phosphorylation has not been described. In this context, it is also important to note that loss-of-function mutations in canonical PINs, even when combined, do not lead to complete inhibition of phototropic bending. It thus appears that several redundant factors regulate this process, with photomorphogenesis and the type of irradiation (pulse vs continuous illumination) contributing to light-controlled directional organ growth as well.^35^ ^,^ ^51-53^

General volatile anesthetics (GVA) are a group of structurally unrelated chemicals known for their ability to block the generation and transmission of electrical signals in animal neurons, thereby causing amnesia, analgesia, and unconsciousness.^54^ A lesser-known fact is that GVA can also affect the plants and many other living organisms.^55^ ^,^ ^56^ This is due to the lipophilic properties of GVA, which can dissolve into the phospholipid bilayer of biological membranes or non-specifically bind to the lipophilic parts of proteins, thereby disrupting a range of physiological processes.^54^ ^,^ ^57-60^ Among the most striking examples of anesthesia effects on plants ranks the inhibition of touch-induced fast movements, such as the folding of leaves of the sensitive plant (*Mimosa pudica*) or the closing of the traps of the Venus flytrap (*Dionaea muscipula*), which were first described in the 19^th^ century.^55^ ^,^ ^61-64^ Only recently, it was found that these effects are caused by GVA's ability to inhibit plant electrical and calcium (Ca^2+^) signaling, which trigger these fast movements.^64-66^ However, recent research has revealed that the effects of anesthetics on plants are much broader and pleiotropic. Plants under anesthesia exhibited disrupted response to light (including photomorphogenesis), wounding responses, and germination, among other effects.^64^ ^,^ ^65^ ^,^ ^67^ ^,^ ^68^ On the cellular level, several studies have shown that GVA can induce a slight cytosolic Ca^2+^ spike and reactive oxygen species production (ROS), thereby triggering diverse responses, including the expression of stress-related genes (e.g., heat shock proteins^64^ ^,^ ^69^ ^,^ ^70^). Additional research has shown that GVA disrupts vesicular trafficking in plant cells,^64^ a process essential for many physiological processes, such as auxin redistribution, which controls tropic growth responses. Like in animals, these drug-induced effects are reversible in plants following non-lethal GVA exposure, indicating that the observed responses reflect a transient, functional modulation of physiological processes rather than irreversible cellular damage or structural injury.^56^ ^,^ ^64^ ^,^ ^65^ ^,^ ^67^

In light of the key role of protein sorting and distribution in the regulation of phototropic organ bending, together with the demonstrated effects of GVA on such protein sorting, we investigated BL-induced hypocotyl phototropism in *A. thaliana* in response to diethyl ether. For this, we determined the kinetics of phototropic organ bending and analyzed the initial events of phototropic signaling, as well as the dynamics in intracellular PIN3 distribution. Our findings have revealed previously unknown effects of diethyl ether on differential auxin distribution and PIN3 sorting in response to unilateral illumination.

## Materials and methods

### Plant material and culture conditions

We used 5-day-old *A. thaliana* Columbia-0 wild-type and transgenic *A. thaliana*: *DR5:GUS*, *PIN3:PIN3-YFP,* and *LTI6B:LTI6B-GFP* reporter lines in our experiments.^71-73^ Seeds were kept in distilled water in the dark at 4 °C overnight for stratification. Before sowing, the seeds were sterilized by immersion in a 1% sodium hypochlorite solution for 15 min. Seedlings were grown on Murashige-Skoog (MS) medium^74^ (Duchefa, Netherlands) in square Petri dishes. Germination was induced by illumination at 100 μmol m^−2^ s^−1^ PAR in an AR75L growth chamber (Percival-Scientific, USA) for approximately 5 h. Afterwards, the nutrient plates were placed in the dark to obtain the etiolated phenotype of the seedlings. The temperature was set to 22 ± 1 °C.

### Analysis of phototropic and gravitropic responses

We investigated the effect of diethyl ether on the hypocotyl phototropism of 5-day-old *A. thaliana* etiolated and de-etiolated seedlings in concurrent experimental setups. For de-etiolation, the seedlings in the plates were transferred to light in the growth chamber, 24 h before the induction of phototropism (100 μmol m^−2^ s^−1^ PAR; AR75L Percival-Scientific, USA), whilst etiolated seedlings remained in the dark. The next day, the plates containing etiolated and de-etiolated seedlings were opened, the seedlings were photographed, and the plates were placed in stands within transparent, sealable plastic bags (low-density polyethylene, LDPE). A beaker containing an appropriate volume of diethyl ether (0.73 mL of liquid diethyl ether per 1 L of air; Sigma-Aldrich, USA) was placed in the bag, which was then immediately closed. Diethyl ether evaporated to a final concentration of 15% within 2 h, to induce responses in plants.^64^ ^,^ ^67^ After incubation, seedlings were exposed to unilateral low-intensity BL (LBL (1 μmol m^−2^ s^−1^; λ_max_ = 460 nm). After 8 h, the seedlings were removed from the diethyl ether atmosphere, photographed under green LED light, and returned to the front of a unilateral LBL source. Recovered seedlings were photographed under green LED light 16 h and 40 h after removal of diethyl ether (after 24 h and 48 h of unilateral irradiation in total). Control seedlings were placed in the same LDPE bags without diethyl ether and photographed at the same time points as the etherized seedlings. The images were analyzed in ImageJ software (NIH, USA), and hypocotyl angles and relative hypocotyl lengths were compared. The relative hypocotyl length was calculated for each seedling according to the following formula: $\mathrm{relative hypocotyl length}=l_{i}/l_{0}$ , where *l*_*i*_ denotes the hypocotyl length at time *i,* and *l*_*0*_ denotes the hypocotyl length at the beginning of the experiment. The correlation between phototropic bending and hypocotyl elongation was also calculated as Pearson’s correlation coefficient from all measured seedlings combined. Three independent experiments were performed with 5–15 seedlings per condition and experiment (Supplementary Fig. S1).

In addition to phototropism, the effect of diethyl ether on gravitropism was also tested. In the gravitropic experiments, only 5-day-old etiolated *A. thaliana* seedlings were used. Gravitropic experiments were conducted in the same manner as the phototropic experiments, except that instead of illumination with unilateral LBL after etherization, the plates with seedlings were rotated by 90 ° to induce gravitropic stimulation. Seedlings were photographed at 0 h, 8 h, and 24 h after gravistimulation. After 8 h of gravitropic stimulation, the plants were removed from diethyl ether and allowed to recover. Gravitropic bending and relative hypocotyl length were measured and evaluated as for phototropism, using ImageJ software. Two independent experiments were performed with 15–20 seedlings per condition.

### Protein isolation, SDS-PAGE, and western blotting

To determine the phosphorylation status of PHOT1 and NPH3, which serve as a markers of early phototropic events, we investigated the possible effect of diethyl ether using protein extraction, followed by SDS-PAGE and Western blot analysis. Experiments were conducted with etiolated etherized and etiolated control 5-day-old seedlings (50 seedlings per condition), which were harvested and frozen in liquid nitrogen before and 20 min after unilateral LBL illumination. Plant material was homogenized using a cooled mortar and pestle. For total protein isolation, homogenized material was transferred to plastic tubes containing extraction buffer (modified SDS sample buffer^75^) as published in Sullivan et al.^76^, with several modifications of our own: the buffer contained 125 mM Tris-HCl (pH 6.8), 2% SDS, 2% DTT, 10 mM NaF as phosphatase inhibitor, and protease inhibitors (Set VI, Calbiochem, Germany). Samples were heated for 5 min at 90 °C, centrifuged at 16000 x g for 10 min, and the pellet was discarded. The following analyzes were done with the supernatant. The concentration of total soluble proteins was determined spectrophotometrically at λ = 562 nm (Synergy MX, BioTek Instruments, USA) using the Bicinchoninic Acid Kit for Protein Determination (Sigma-Aldrich, USA). Next, equal amounts of proteins were mixed in a ratio of 3:1 (v/v) with loading buffer (modified SDS sample buffer,^75^) with a final concentration in the sample: 62.5 mM Tris-HCl (pH 6.8), 2.5% SDS, 10% glycerol, 50 mM DTT, and 0.002% bromophenol blue. Proteins were separated in 6.5% (v/v) SDS-PAGE gel^77^ and transferred to a nitrocellulose membrane (Bio-Rad, USA) in the Trans-Blot Turbo System (Bio-Rad, USA). Membranes were then stained with Ponceau-S to check the correct protein transfer. Membranes were blocked for 1 h in TBS-T containing 5% BSA and then incubated with the primary antibody overnight at 4 °C. Antibodies against PHOT1 (1:80000^78^) phosphorylated S350 of PHOT1 (1:80000;^76^), NPH3 (1:10000^79^) and phosphorylated S744 of NPH3 (1:20000^29^) were kindly provided to us by John Christie (University of Glasgow, UK). After washing, membranes were incubated for 1 h in the secondary antibody (1:10000; goat anti-rabbit IgG (H + L)-horseradish peroxidase conjugate; Bio-Rad, USA). Immobilon Western chemiluminescent HRP substrate (Millipore, USA) was used for immunodetection on an Amersham Imager 600 (GE Healthcare Life Sciences, USA). Three independent biological replicates, each with 1–2 technical replicates, were performed.

### Auxin distribution analysis with GUS assay

For analysis of auxin distribution in response to unilateral LBL and diethyl ether, we used transgenic *A. thaliana DR5:GUS* 5-day-old seedlings in the experimental setup as described above.^72^ Seedlings were harvested at 0 h, 8 h, and 24 h after LBL irradiation, and etherized seedlings were taken out of a diethyl ether atmosphere after 8 h of irradiation to recover. Seedlings were harvested into 6-well plates with GUS staining solution containing 0.2% X-Gluc (10% X-Gluc in DMSO; Thermo Fisher Scientific, USA), 0.1 M Na-phosphate buffer (pH 7.0), 2 mM K_4_[Fe(CN)_6_], 2 mM K_3_[Fe(CN)_6_] and 0.1% (v/v) Triton. Seedlings underwent 20-min vacuum infiltration in the staining solution and were incubated for 48 h at 37 °C in the dark. Subsequently, the seedlings were transferred to 70% EtOH, placed on slides, and observed under a stereomicroscope Olympus SZX16 (Japan). The number of seedlings with a staining gradient on the shaded vs illuminated side of the hypocotyl was scored. The percentage representation of this signal gradient in the population was then compared between individual sample types. We performed 5–9 biological replicates per condition, each with 3–17 seedlings per condition and experiment.

### PIN3 localization analysis with fluorescence confocal microscopy

For analysis of the abundance and localization of the PIN3 auxin efflux carrier, which is involved in light-dependent auxin redistribution, we used 5-day-old transgenic *PIN3:PIN3-YFP* seedlings.^73^ Experiments were done with etiolated control and etherized seedlings, grown in the same conditions as described above. Confocal analysis was obtained before and after 8 h of unilateral LBL illumination. Since PIN3 polarization during phototropism occurs quite rapidly, we omitted analysis after extended incubation periods. Instead, the recovery of PIN3 polarization was analyzed at 2, 4, and 6 h after diethyl ether removal. As a control of membrane integrity, the *LTI6B:LTI6B-GFP* plasma membrane protein marker line was employed,^71^ and analyzed under the same conditions as PIN3-YFP. CLSM images were generated using a Leica SP5 confocal microscope (Leica Microsystems, Germany). Fluorescence markers were excited using an argon laser. The signal intensity was analyzed using ImageJ software (NIH, USA). For each analyzed cell, the fluorescence intensity (FI; arbitrary units) was measured on the lateral membrane (*FI*_*LATERAL*_), on the apical membrane (*FI*_*APICAL*_), and in the cytosol (*FI*_*CYTOSOL*_). From these values, the relative intensity for the lateral membrane was then calculated as ${L=\mathrm{FI}}_{\mathrm{LATERAL}}/{\mathrm{FI}}_{\mathrm{CYTOSOL}}\times 100$ ; for the apical membrane as ${A=\mathrm{FI}}_{\mathrm{APICAL}}/{\mathrm{FI}}_{\mathrm{CYTOSOL}}\times 100$ ; and the L/A ratio as $L/A={\mathrm{FI}}_{\mathrm{LATERAL}}/{\mathrm{FI}}_{\mathrm{APICAL}}\times 100$ (figure explaining the measured areas and the derived values of relative fluorescence intensities is available as Supplementary Fig. S2). The number of independent experiments performed was 1–4, with 3–10 individual seedlings per experiment and 10–40 individual endodermal cells analyzed on both sides of the hypocotyl per seedling.

### Statistical analysis

To analyze the relationship between phototropic bending and hypocotyl elongation, Pearson's correlation coefficient was calculated. In further analyzes, Student t-test was used to compare two data sets, and ANOVA with Tukey post hoc test was used to compare three or more data sets. Statistical analyzes were performed in Excel (Microsoft, USA) and Origin (OriginLab, USA).

## Results

### Diethyl ether anesthesia inhibited the phototropic response of A. thaliana hypocotyl

**Figure 1.**
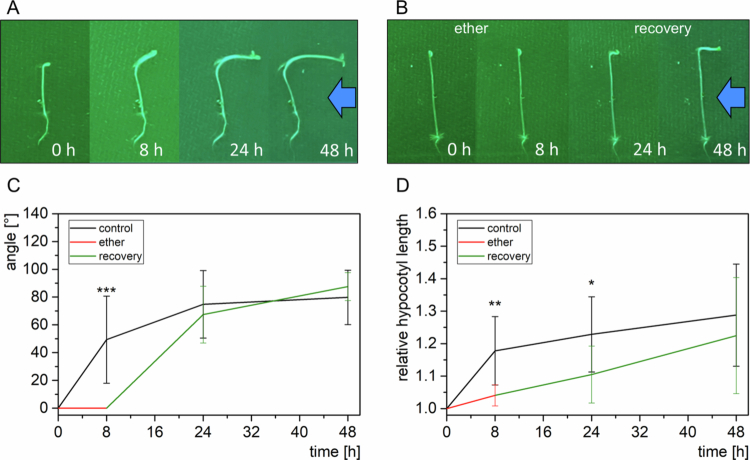
Phototropic response of 5-day-old *A. thaliana* etiolated hypocotyls under diethyl ether anesthesia. Phototropic response of control (A) and etherized (B) seedlings after 0 h (dark), 8 h, 24 h, and 48 h unilateral LBL irradiation (1 μmol m^−2^ s^−1^). Etherized seedlings were moved from a diethyl ether atmosphere after 8 h of LBL irradiation and allowed to recover (24 h and 48 h depict recovered phototropism). Representative images are shown. The blue arrows depict the direction of LBL. (C) Phototropic bending and (D) relative hypocotyl length of control (black lines), etherized (red lines), and recovered (green lines) seedlings. Data depict means ± SD from a single representative experiment (*n* = 5–15). Significant differences between control and etherized seedlings evaluated by Student t-test are denoted by asterisks (* = *P* < 0.05; ** = *P* < 0.01; *** = *P* < 0.001).

When 5-day-old etiolated Col-0 wild-type seedlings were exposed to unilateral LBL, a pronounced phototropic curvature of the hypocotyl was evident after 8 h of irradiation, reaching an average bending angle of approximately 50°. The curvature continued to increase over time, with seedlings displaying stronger bending after 24 h and reaching a plateau at around 48 h, with an average bending angle of approximately 90° (Figure 1; Supplementary Fig. S3). These data indicate a rapid and robust phototropic response under continuous unilateral LBL stimulation.

In contrast, seedlings exposed to a diethyl ether atmosphere exhibited a complete loss of hypocotyl phototropic responsiveness. After 8 h of unilateral LBL irradiation, their average hypocotyl bending remained close to 0°, demonstrating a near-total suppression of directional growth (Figure 1; Supplementary Fig. S3). This effect was reversible, as the phototropic response was recovered when the seedlings were removed from a diethyl ether atmosphere. While the phototropic response of recovered seedlings was still less pronounced than that of control seedlings after 24 h (16-h recovery), there was no significant difference after 48 h (40-h recovery), when compared to untreated controls (Figure 1; Supplementary Fig. S3). The restoration of bending implies that diethyl ether does not cause irreversible damage to the phototropic machinery but instead only transiently interferes with its function.

In addition to phototropic bending, we quantified relative hypocotyl elongation to determine whether growth inhibition might account for the impaired bending. The elongation was attenuated under diethyl ether, but it was not completely abolished, as the hypocotyl still grew to a limited extent (Figure 1; Supplementary Fig. S3). Importantly, correlation analysis revealed only a weak relationship between inhibition of hypocotyl elongation and suppression of phototropic curvature (r = 0.24; Supplementary Fig. S4). This lack of a strong correlation suggests that the inhibition of bending cannot be explained solely by reduced growth rates. Instead, it appears that diethyl ether disrupts components of the phototropic response pathway, rather than generally inhibiting cell elongation.

When comparing etiolated and de-etiolated seedlings, we found differences only in the hypocotyl elongation rate. Specifically, diethyl ether-induced inhibition of hypocotyl elongation was significant in etiolated plants but not in deetiolated plants (Figure 1, Supplementary Fig. S3). In contrast, no significant differences between etiolated and de-etiolated seedlings were found with respect to phototropic bending under any of the tested conditions (Figure 1; Supplementary Fig. S3). Based on the absence of differential effects on phototropic curvature, and given the clearer elongation phenotype in etiolated seedlings, we therefore conducted all subsequent analyzes exclusively using etiolated material.

In addition, we analyzed the effect of diethyl ether on the hypocotyl gravitropic responses of 5-day-old etiolated *A. thaliana* seedlings, as this also depends on a lateral auxin gradient uncoupled from phototropic signaling. In control plants after 8 h, the apparent gravitropic bending was, on average, 30°, whereas in plants under the influence of diethyl ether, it was absent (Supplementary Fig. S5). This effect was again fully reversible, as 16 h after removal from the diethyl ether atmosphere (24-h gravistimulation), the angle between control and etherized plants was not statistically distinguishable, averaging around 50°. As in the phototropic experiments, we also analyzed relative hypocotyl elongation and confirmed that, even under the influence of diethyl ether, hypocotyls elongate to a limited extent (Supplementary Fig. S5). These data indicate that the effect of diethyl ether is not limited to the phototropic response but, more generally, affects growth responses involving differential cell elongation mediated by the transient formation of an auxin concentration gradient.

### Early phototropic signaling was unaffected by diethyl ether

To determine whether the suppression of phototropic hypocotyl curvature by diethyl ether involves adjustments in early signal perception, we analyzed key components of the phototropic pathway at the protein level. Specifically, we performed SDS–PAGE and Western blot analyzes to examine the abundance and phosphorylation status of PHOT1 and NPH3 in 5-day-old etiolated seedlings before and after 20 min of unilateral LBL irradiation.

**Figure 2.**
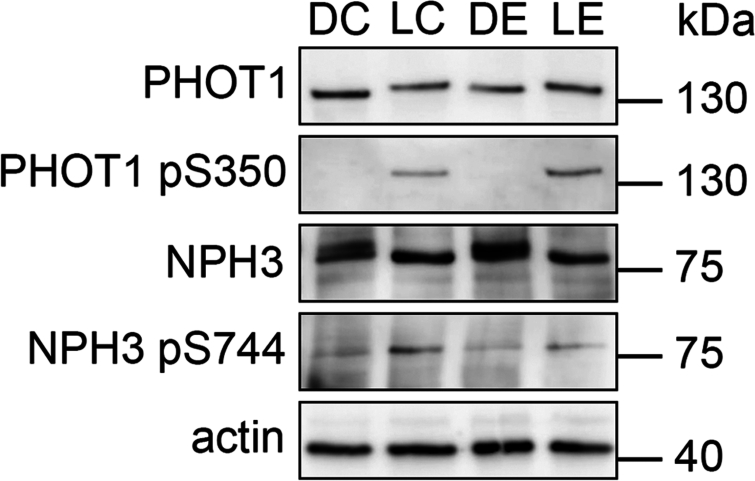
Western blot analysis of the LBL-dependent phosphorylation status of PHOT1 and NPH3 under diethyl ether anesthesia in 5-day-old *A. thaliana* etiolated hypocotyls. (DC) control seedlings in the dark, (LC) control seedlings after 20-min LBL irradiation (1 μmol m^−2^ s^−1^), (DE) etherized seedlings in the dark, (LE) etherized seedlings after 20-min LBL irradiation. Blots were probed with anti-PHOT1, anti-NPH3 and phospho-specific pS350 for PHOT1 and pS744 for NPH3. An anti-actin antibody was used as a loading control. Representative blots are shown from three independent experiments (*n* = 3).

In untreated control seedlings, irradiation induced the molecular responses that have been described earlier.^17^ ^,^ ^27^ ^,^ ^29^ PHOT1 protein levels were slightly reduced following light exposure, and the protein displayed an electrophoretic mobility shift toward higher molecular weights (Figure 2), consistent with light-induced autophosphorylation. This observation was corroborated by the analysis of phosphorylation at residue S350, which occurred exclusively in irradiated samples (Figure 2). These results confirm proper activation of PHOT1 upon LBL stimulation. Analysis of NPH3 revealed complementary light-dependent changes, as the NPH3 bands shifted to lower molecular weight in irradiated samples demonstrating an overall protein dephosphorylation, while a S744 in the C-terminal 14-3-3 binding motif of NPH3 remained phosphorylated, indicating formation of the active NPH3 form (Figure 2). Together, these data demonstrate that early phototropic signaling events, with PHOT1 activation and NPH3 dephosphorylation/reorganization, are accurately induced under our experimental conditions.

When comparing these light-dependent modifications of PHOT1 and NPH3 in control and ether-treated seedlings, no striking differences could be detected. Both the irradiation-induced mobility shifts and the site-specific phosphorylation patterns were unaltered upon diethyl ether exposure (Figure 2). Thus, in contrast to the observed suppression of phototropic curvature, early photoreceptor activation and initial signal transduction events remained functional.

### Auxin redistribution to the shaded side of the hypocotyl was disturbed in etherized seedlings

**Figure 3.**
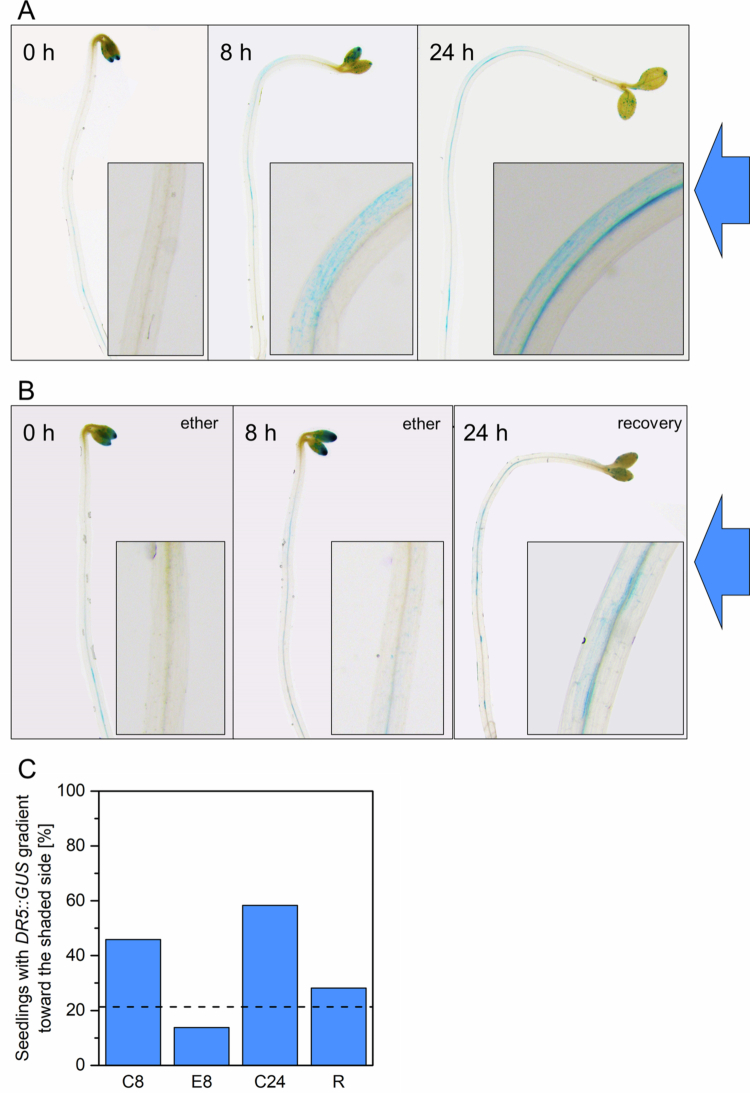
Auxin responses to unilateral LBL (1 μmol m^−2^ s^−1^) in etiolated hypocotyls of 5-day-old transgenic *DR5:GUS A. thaliana* seedlings. (A) representative control and (B) etherized seedlings after 0 h (dark), followed by 8 h and 24 h unilateral LBL irradiation. Etherized seedlings were moved from a diethyl ether atmosphere after 8 h of LBL irradiation and allowed to recover (24 h time point depicts recovered phototropism). *DR5:GUS* reporter activity is visualized as blue precipitate. The blue arrows represent the direction of the LBL. (C) Graph summarizing the percentage of seedlings from the entire analyzed population (*n* = 27–71) exhibiting a GUS signal gradient toward the shaded side of the hypocotyl after 8 h (C8, E8) and 24 h (C24, R) of unilateral LBL irradiation; R indicates seedlings recovered from diethyl ether treatment; the dashed line indicates the percentage of seedlings from the dark (C0 and E0 combined) with the GUS signal gradient in the hypocotyl.

To assess whether the inhibition of phototropic curvature under diethyl ether coincides with impaired formation of an auxin gradient in etherized hypocotyls, we analyzed 5-day-old *DR5:GUS* reporter seedlings as a sensitive readout for auxin-responsive gene expression.^72^ Staining was predominantly detected in the cotyledons, the apical hook, and the basal vascular tissues. These staining patterns were consistent across treatment conditions, indicating that auxin distribution and reporter activity were not affected by diethyl ether in the absence of directional light. Under dark conditions, most control and etherized seedlings had symmetrically distributed GUS staining on both sides of the hypocotyl (approximately 80% of all seedlings regardless of treatment; Figure 3). On the other hand, in the remaining seedlings from the dark conditions, GUS staining was asymmetric without a known reason (Figure 3).

Following 8 h of unilateral LBL irradiation, all control seedlings exhibited phototropic bending, with approximately half displaying a clear lateral gradient, with stronger GUS staining on the shaded side of the hypocotyl (Figure 3). This asymmetry became more pronounced after 24 h of irradiation, coinciding with the increase in phototropic curvature observed, which is consistent with the established model of phototropism, in which lateral auxin redistribution toward the shaded flank promotes differential cell elongation and bending. In striking contrast, etherized seedlings with their complete absence of hypocotyl curvature failed to establish a robust GUS signal gradient after 8 h of unilateral irradiation and the proportion of seedlings displaying the shaded-side gradient was strongly reduced (the proportion was not higher than random under dark conditions; Figure 3). Upon removal from the diethyl ether atmosphere, both asymmetric *DR5:GUS* staining and phototropic bending gradually recovered, although the frequency and intensity of this asymmetry remained somewhat lower than in untreated controls at comparable time points (Figure 3). Overall, impairment in GUS signal asymmetry together with its partial restoration upon compound removal, supports a hypothesis in which diethyl ether reversibly interferes with the redistribution of auxin required for directional growth.

### Light-dependent PIN3 localization was affected by diethyl ether

**Figure 4.**
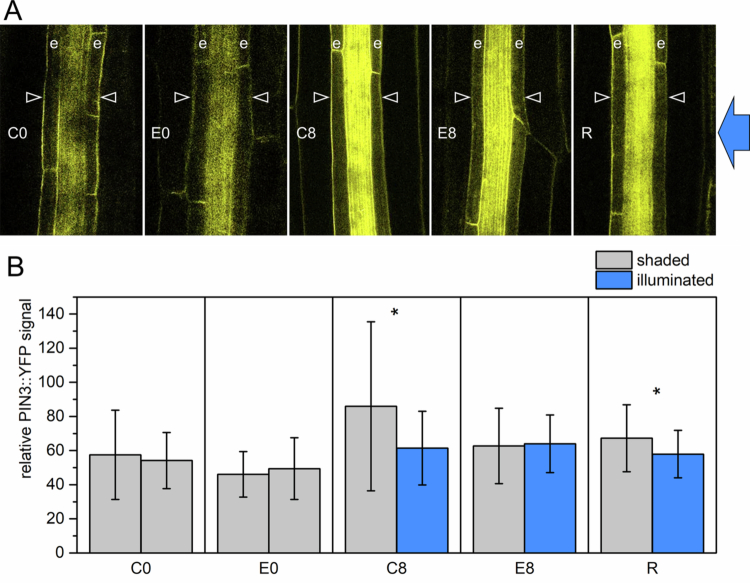
PIN3-YFP signal distribution in response to unilateral LBL (1 μmol m^−2^ s^−1^) in etiolated upper hypocotyl endodermal membranes of 5-day-old transgenic *PIN3:PIN3-YFP A. thaliana* seedlings. (A) Representative images showing YFP signals (yellow) in the endodermis (e). The blue arrow indicates the direction of the LBL. Arrowheads highlight the PIN3-YFP signal polarity at the lateral PM domains. C0: untreated and non-illuminated seedling; E0: etherized non-illuminated seedling; C8: control after 8 h of unilateral LBL irradiation; E8: etherized seedling after 8 h of unilateral LBL irradiation (10 h etherization); R: recovered seedling, 4 h after removal from the diethyl ether atmosphere and continuous unilateral LBL irradiation. (B) Plots displaying the average PIN3-YFP signal intensity (L/A ratio) on shaded and illuminated lateral endodermal PM domains; means ± SD. The number of analyzed cells on each side is *n* = 15–31. Significant differences between control and etherized seedlings evaluated by Student t-test are denoted by asterisks (* = *P* < 0.05). Relative units were set as: ${L/A=\mathrm{FI}}_{\mathrm{lateral}}\;{\mathrm{FI}}_{\mathrm{apical}}\times 100$ . Boxplots representing PIN3 signal intensities (L value, A value and L/A ratio) of the total amount of C8 and E8 cells scored are available as Supplementary Fig. S6.

To further dissect the mechanism underlying the impaired auxin redistribution in diethyl ether-treated seedlings, we examined the subcellular localization of PIN3, which is required for lateral auxin redistribution during phototropism in *A. thaliana.* ^43^ ^,^ ^44^ Consistent with previous reports, PIN3-YFP in dark-grown hypocotyls was symmetrically distributed along the lateral membranes of endodermal cells in the upper hypocotyl region. Upon exposure to unilateral LBL, PIN3 signals underwent redistribution, with pronounced signals at the outer lateral PM domains of endodermal cells on the shaded side of the hypocotyl, reflected in a significant increase in the lateral-to-apical (L/A) fluorescence intensity ratio on the shaded side. (Figure 4; Supplementary Fig. S6). This heterogeneity was clearly visible after 8 h of unilateral LBL irradiation. In striking contrast, etherized seedlings failed to establish this characteristic PIN3 polarity. Instead, the PIN3-YFP signal remained evenly distributed across the endodermal PM domains, hinting at deficiencies in rerouting processes. Moreover, overall PM-associated PIN3 signal intensity represented by the A value (both illuminated and shaded sides) was reduced in the presence of diethyl ether, suggesting either diminished membrane stability or altered protein trafficking dynamics under anesthetic treatment (Figure 4; Supplementary Fig. S6). As observed for phototropic hypocotyl bending, diethyl ether effects on PIN3 distribution were reversible, with PIN3-YFP signal asymmetry (L/A ratio) in unilaterally illuminated hypocotyls detected 4 h after removal of diethyl ether (Figure 4).

To assess whether diethyl ether effects were specific to PIN3 or reflected a more general disturbance of membrane-associated proteins, we analyzed the localization of the PM marker LTI6B-GFP. In untreated seedlings, LTI6B-GFP displayed uniform membrane distribution and showed no significant differences in fluorescence intensity between illuminated and shaded sides (L/A value; Supplementary Fig. S7). In etherized LTI6B-GFP seedlings, a moderate reduction in PM signals at the apical PM domain (A value) was observed for LTI6B-GFP, whereas no differences were observed for the other measured parameters (Supplementary Fig. S7). Thus, diethyl ether treatment appears to cause subtle changes in the distribution or abundance of LTI6B-GFP as well; however, these do not resemble the pronounced differences observed for PIN3-YFP, potentially reflecting an increased sensitivity of dynamically redistributed PIN3 to inhibitory diethyl ether effects.

## Discussion

In this study, we demonstrated that diethyl ether strongly inhibits the phototropic response of hypocotyls to unilateral LBL in *A. thaliana*, and that this effect likely arises from two distinct mechanisms (Figure 1; Supplementary Fig. S3). First, diethyl ether partially suppresses hypocotyl elongation. Second, the compound specifically interferes with the phototropic bending response. The inhibition of hypocotyl growth potentially involves multiple factors. One plausible scenario involves the induction of physiological stress responses, consistent with previous reports that diethyl ether elicits stress responses in plants (e.g., increased cytoplasmic Ca^2+^ concentration [Ca^2+^]_cyt_, ROS production, or accumulation of heat shock proteins^64^ ^,^ ^70^) leading to negative effects on overall plant growth. Indeed, elevated [Ca^2+^]_cyt_ may induce hypocotyl growth inhibition.^80^ A more specific effect could be mediated through modulation of PIN protein abundance at the PM, as our data indicate quantitative alterations in PM-localized PIN3 under diethyl ether treatment (Supplementary Fig. S6). Attenuation of PIN3 abundance, for example, has earlier been demonstrated to mitigate hypocotyl elongation growth.^81^ Apart from PIN3, diethyl ether could also affect the sorting and distribution of further canonical PIN proteins, potentially contributing to overall defects in hypocotyl elongation.

Whilst etherized hypocotyls continued to elongate to a limited extent, phototropic bending was almost completely suppressed by diethyl ether treatment (Figure 1; Supplementary Figs. S3, S4), highlighting an additional, specific inhibitory effect on the phototropic signaling pathway. Our experiments, therefore, first focused on LBL responses, primarily mediated by the photoreceptor PHOT1.^11^ Upon blue light activation, PHOT1 undergoes autophosphorylation and triggers modifications in the phosphorylation status of interacting proteins, such as NPH3, with consequences for localization and protein half-life.^8^ ^,^ ^20^ ^,^ ^21^ ^,^ ^24^ Diethyl ether, like other anesthetics, interferes with the integrity of biological membranes by altering their physicochemical properties,^58^ ^,^ ^82^ which could theoretically affect membrane-associated proteins such as PHOT1. Moreover, some ether-derived anesthetics have been reported to alter protein phosphorylation profiles in animal systems,^83^ suggestive of related effects on protein kinase-mediated signaling in plants. Nevertheless, analysis of PHOT1 and NPH3 abundance and phosphorylation did not reveal any striking differences between control and etherized seedlings (Figure 2), indicating that early phototropic signaling proceeds without fundamental disruption under diethyl ether. Diethyl ether is known to increase [Ca^2^⁺]_cyt_ and affect ion channels and cytosolic Ca^2^⁺ dynamics.^66^ ^,^ ^67^ ^,^ ^69^ ^,^ ^70^ PHOT1 activation has also been reported to induce an increase in the [Ca^2^⁺]_cyt_; however, this Ca^2^⁺ spike does not appear to be required for LBL-induced, PHOT1-regulated phototropism.^80^ This contrasts with high-intensity BL phototropism, which is regulated by PHOT2 and involves Ca^2^⁺ signaling.^84^ Thus, the specific inhibition of PHOT1-dependent phototropic signaling by diethyl ether observed in our study cannot be readily attributed to altered Ca^2^⁺ signaling, despite the known effects of diethyl ether on [Ca^2^⁺]_cyt_.

Our observations indicating that diethyl ether does not inhibit early signaling events are further supported by the hypocotyl gravitropism experiments (Supplementary Fig. S5). Gravitropic hypocotyl bending, which is independent of early PHOT-mediated signaling but also requires establishment of a lateral auxin gradient,^85^ ^,^ ^86^ was similarly affected by diethyl ether, pointing to auxin redistribution as a likely target of anesthetic interference.

Auxin redistribution to the shaded side of organs is a key event in phototropic bending.^44^ ^,^ ^87^ In control seedlings, auxin-responsive *DR5:GUS* reporter analysis revealed a clear gradient of staining toward the shaded side after unilateral irradiation (Figure 3), consistent with the established model in which lateral auxin accumulation drives differential cell elongation. In etherized seedlings, however, this gradient was substantially reduced, though not entirely abolished. Recovery from diethyl ether partially restored both phototropic bending and formation of the auxin gradient, although not to the extent observed in untreated controls (Figure 3). These observations indicate that diethyl ether reversibly disrupts light-induced lateral auxin redistribution that occurs downstream of light perception. It is important to note that the *DR5:GUS* system has its limitations, and in our experiments, we observed both non-irradiated plants with an asymmetric GUS signal distribution and phototropically bent control plants that, by contrast, lacked a GUS signal gradient in the hypocotyl, possibly due to gene silencing. Nevertheless, given the volume of our data and the clear trend, we consider this analysis sufficiently robust for our investigations.

A well-established pathway underlying lateral auxin gradient formation in *A. thaliana* hypocotyls involves the light-dependent redistribution of the auxin efflux carrier PIN3 in endodermal cells.^43^ ^,^ ^44^ In control seedlings, PIN3-YFP accumulated preferentially on lateral PM domains of endodermis cells at the shaded side of the hypocotyl, whereas etherized seedlings showed homogeneous PIN3 distribution and a reduced overall membrane signal (Figure 4; Supplementary Fig. S6). Diethyl ether thus appears to affect the coordinated sorting and redistribution of PIN3 in response to the light stimulus, which is consistent with the compound’s demonstrated effects on vesicular sorting and membrane integrity.^64^ ^,^ ^65^ ^,^ ^70^ Strikingly, analysis of the apolarly localized PM marker LTI6B-GFP revealed changes in its PM signal distribution under diethyl ether treatment, less pronounced than those observed for PIN3 (Supplementary Fig. S7). This indicates that diethyl ether exerts more general effects on the fate of PM proteins. However, the elevated sensitivity of PIN3 distribution to diethyl ether suggests that PM proteins undergoing highly dynamic localization adjustments are particularly vulnerable to diethyl ether-mediated disruption of sorting processes. This scenario would also explain the distinct anesthetic effects on PIN3-dependent processes in hypocotyls.

**Figure 5.**
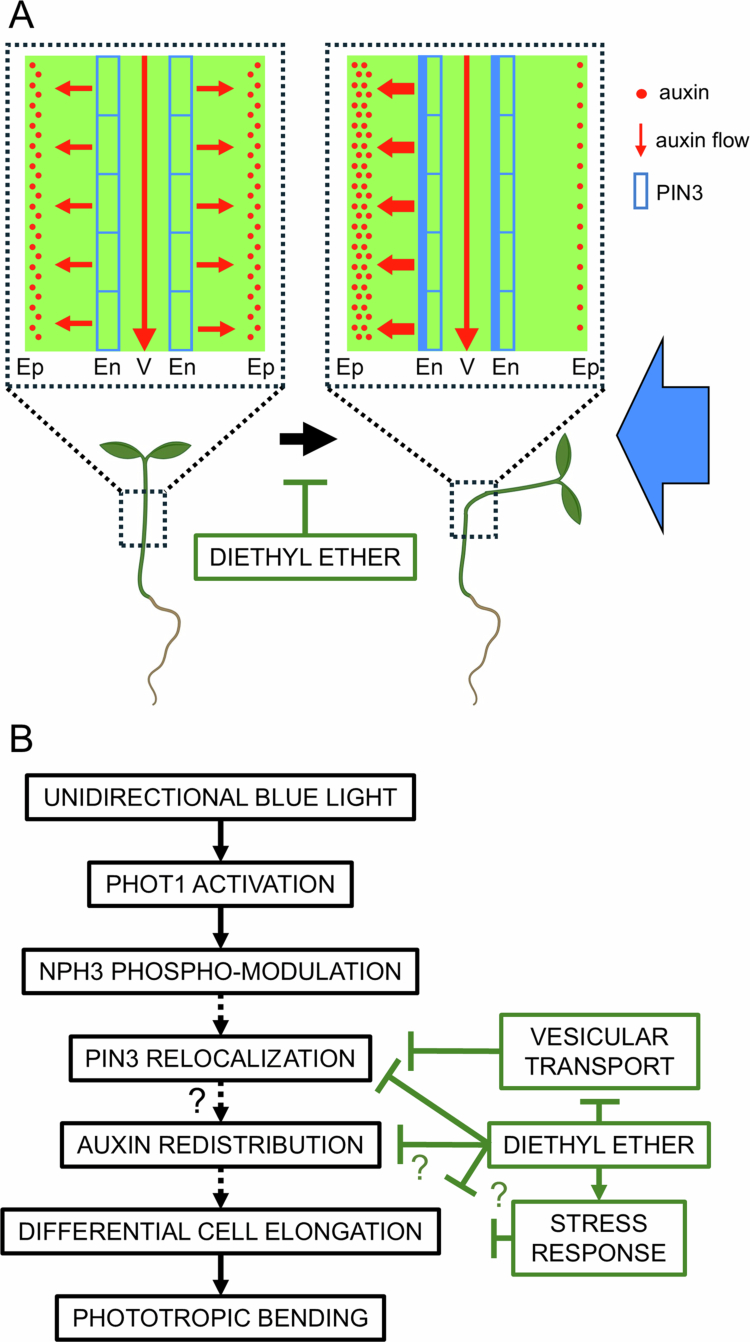
Proposed model of the effect of diethyl ether anesthesia on phototropic response in *A. thaliana*. (A) Scheme of a longitudinal section of the upper hypocotyl portion during phototropic response; Ep: epidermis; En: endodermis; V: vasculature; red dots: auxin; red arrows: direction of auxin flow; blue rectangles: PIN3 distribution on endodermal PM domains; blue arrow: direction of the LBL. (B) Proposed sites of action of diethyl ether in the phototropic response signaling pathway. Early steps of the phototropic signaling pathway appear unaffected by diethyl ether, whereas it interferes with PIN3 distribution control and lateral auxin gradient formation. Alterations in PIN3 distribution in the etherized seedlings might arise from inhibitory effects on vesicular protein transport and/or overall alterations in PM integrity, which might affect PIN3 accumulation in membrane domains. Abolishment of PIN3 polarity likely results in a disturbance of auxin gradient formation. Nevertheless, additional diethyl ether's effects on auxin transport via alternate mechanisms cannot be ruled out. In addition, diethyl ether effects on the overall control of cell elongation, potentially caused by diethyl ether-induced stress response (e.g., Ca^2+^ transient, ROS production, or accumulation of heat shock proteins) might contribute to the phenotypes observed.

Several mechanisms could explain the diethyl ether-induced interference with PIN3 redistribution. PIN3 localization is regulated by the protein kinases PID and D6PK, which control its subcellular localization and activity via reversible protein phosphorylation^46^ ^,^ ^49^ ^,^ ^50^ and via sorting processes that rely on vesicular transport.^45^ ^,^ ^47^ ^,^ ^48^ ^,^ ^81^ ^,^ ^88^ Anesthetics like diethyl ether have been demonstrated to interfere with vesicle recycling in *A. thaliana* roots,^64^ and RNA-seq experiments demonstrated vesicular transport among highly enriched and upregulated GO categories (including e.g., *PID* or *D6PK* among others) in *A. thaliana* leaves.^70^ Indeed, overexpression of *PID* inhibited PIN3 polarity and phototropic bending.^43^ The reduced PM signal of PIN3 and, to a limited extent, of LTI6B in etherized hypocotyls supports a model in which diethyl ether impairs vesicular trafficking, thereby preventing proper PIN3 localization and consequently the establishment of lateral auxin gradients. Although this model appears robust (Figure 5), alternative or additional mechanisms of auxin redistribution need to be considered, including regulation via ABCB19^35^ or proton pumps.^31^ ^,^ ^89^ ^,^ ^90^ Moreover, the contribution of PIN3 redistribution relative to auxin accumulation remains under discussion,^35^ ^,^ ^51^ ^,^ ^52^ as additional auxin transport pathways also contribute to auxin redistribution during phototropism.^23^ ^,^ ^51^ ^,^ ^52^

Taken together, our findings indicate that diethyl ether exerts dual inhibitory effects on hypocotyl phototropism, namely, a general reduction in elongation and an interference with the formation of a lateral auxin gradient required for bending. This effect appears to involve impaired PIN3 redistribution, likely through interference with vesicular trafficking and/or regulatory processes involved, rather than disruption of early photoreceptor activation.

## Conclusion

Our results demonstrate that diethyl ether almost completely suppresses hypocotyl phototropism in *A. thaliana* while allowing limited elongation growth, indicating a specific interference with directional growth signaling rather than a general inhibition of growth. Early phototropic signaling events, namely PHOT1 activation and NPH3 phosphorylation dynamics, remained unaffected under diethyl ether treatment, suggesting that light perception and initial signal transduction are largely intact. In contrast, both *DR5:GUS* reporter analysis and PIN3-YFP imaging revealed a strong disruption of light-induced auxin redistribution and PIN3 re-localization in etherized seedlings. These findings indicate that diethyl ether primarily interferes with the establishment of the lateral auxin gradient required for phototropic bending. A plausible explanation is that diethyl ether perturbs vesicular trafficking or regulatory processes controlling PIN3 localization, thereby preventing its redistribution and subsequent auxin transport toward the shaded side of the hypocotyl. Future work will be necessary to determine whether additional auxin transporters, membrane trafficking components, or regulatory enzymes contribute to this anesthetic sensitivity.

## Supplementary Material

Hřivňacký_Supplementary MH5.docx
