# Supplementary material for: Diethyl ether anesthesia inhibits phototropic response in Arabidopsis thaliana by disrupting auxin redistribution
Source: Plant Signal Behav. 2026 Jul 24;21(1):2700912. doi: 10.1080/15592324.2026.2700912 (PMC13418471; doi:10.1080/15592324.2026.2700912)
Supplement: Hřivňacký_Supplementary MH5.docx [file KPSB_A_2700912_SM5618.docx]

**SUPPLEMENTARY MATERIALS**

Diethyl ether anaesthesia inhibits phototropic response in *Arabidopsis thaliana* by disrupting auxin redistribution

**Martin Hřivňacký^1^, Marek Rác^1^, Tereza Miksteinová^2^, Christian Luschnig^3^, Andrej Pavlovič^1^***

*^1^Department of Biophysics, Faculty of Science, Palacký University, Šlechtitelů 27, Olomouc CZ-77900, Czech Republic*

*^2^Laboratory of Growth Regulators, Faculty of Science, Palacký University and Institute of Experimental Botany, Czech Academy of Sciences, Šlechtitelů 27, Olomouc CZ-77900, Czech Republic*

*^3^Institute of Molecular Plant Biology, Department of Biotechnology and Food Science,* *BOKU University Muthgasse 18, AT-1190 Wien, Austria*

**Author for correspondence: andrej.pavlovic@upol.cz*

*Supplementary figures: 7*

**Supplementary Fig. S1 Experimental setup (A) and timeline (B) of the analyses used.** 4–7-day-old *A. thaliana* seedlings on a square Petri dish were placed into a transparent, resealable plastic bag. A beaker containing a volume of diethyl ether (DE) sufficient to produce an atmosphere of 15 % diethyl ether was placed in the bag, which was then immediately sealed. Diethyl ether was allowed to evaporate for 2 h, then unilateral LBL was switched on. After 8 h, seedlings were removed from diethyl ether and allowed to recover. Photo capture (Ph) for bending analysis was performed 2 h after diethyl ether application (0 h) and at 8, 24, and 48 h after unilateral LBL illumination (8 h, 24 h, 48 h). Seedlings for *DR5::GUS* staining (Gu) were harvested at the same time points, except 48 h. Collecting the seedlings for confocal analyses (Co) was done at similar time points, with a variance of ± 2 h allowing for real-time analysis of PIN3-YFP signal distribution. Seedlings for SDS-ELFO & western blot analysis (El) were harvested 20 min before and after LBL illumination. In all aforementioned analyses, control seedlings were harvested/studied at the same times as the etherised seedlings. Created with BioRender.com.


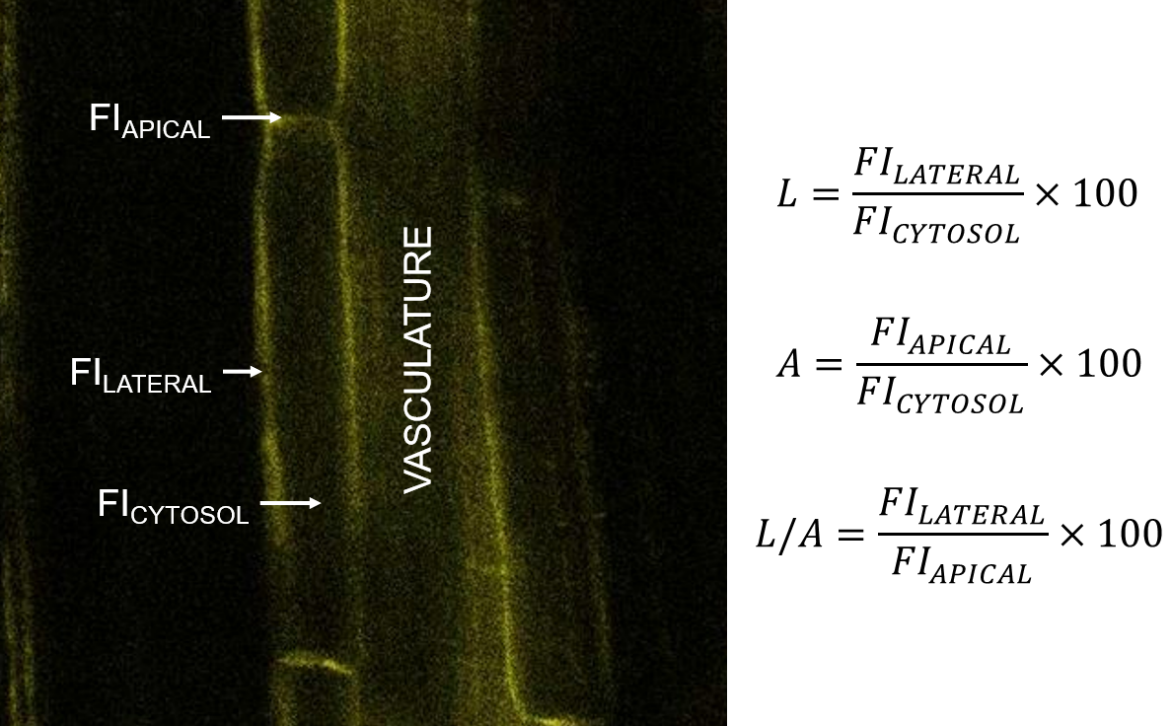


**Supplementary Fig. S2 Specification of the measured sections used to determine fluorescence intensities in endodermal cells, along with the values derived from them.** In our experiments, the L/A ratio was decisive for assessing the distribution of the target protein between the illuminated and non-illuminated sides. At the same time, the A value was crucial for determining the relative amount of the target protein on the PM, and the L value had a supporting role.


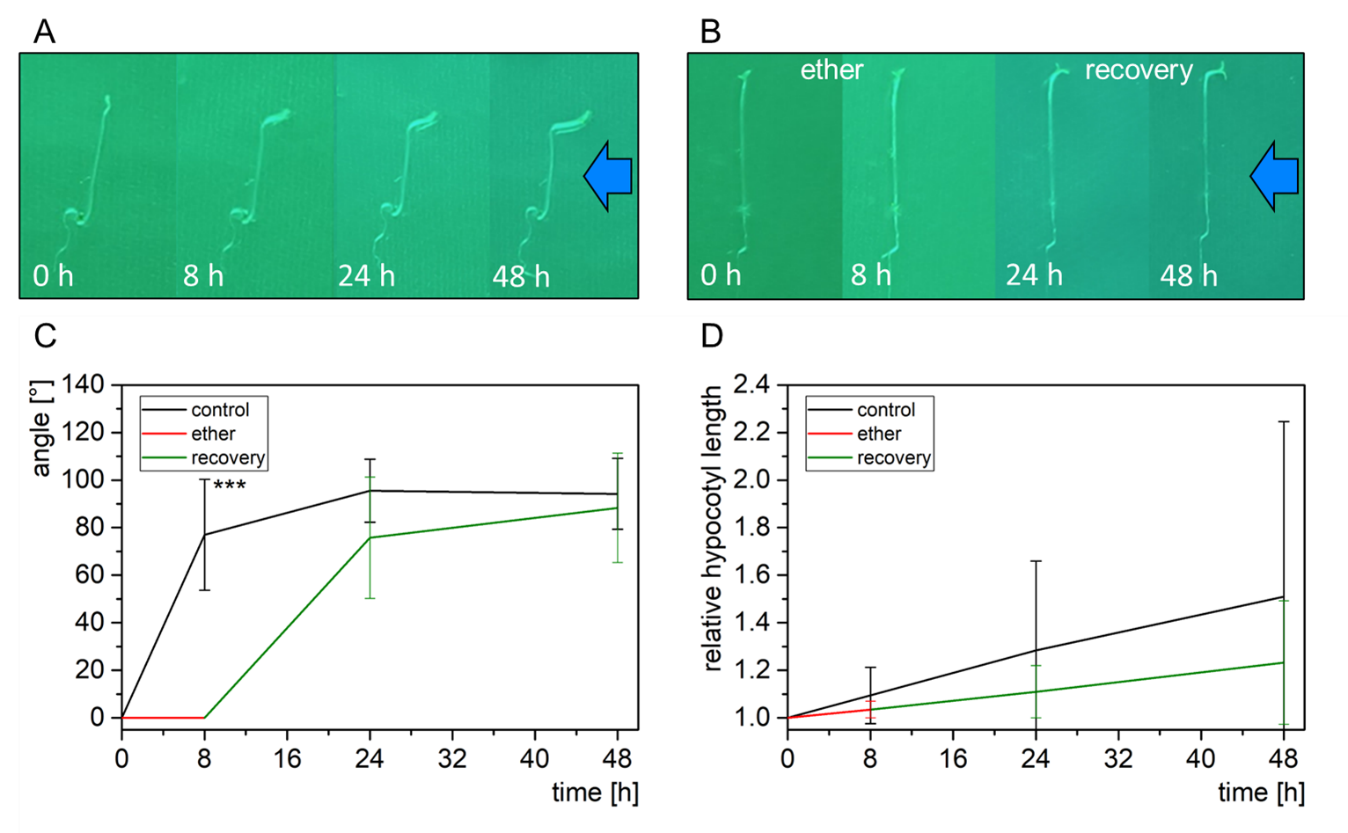


**Supplementary Fig. S3 Phototropic response of 5-day-old *A. thaliana* de-etiolated hypocotyls under diethyl ether anaesthesia.** Phototropic response to unilateral LBL (1 μmol m^-2^ s^-1^) of control (A) and etherised (B) seedlings (removed from diethyl ether after 8 h for recovery). Representative images are shown. The blue arrows depict the direction of LBL. (C) Phototropic bending and (D) relative hypocotyl length of control (black lines), etherised (red lines), and recovered (green lines) seedlings. Data depict means ± SD from a single representative experiment (n = 5–15). Significant differences between control and etherised seedlings evaluated by Student t-test are denoted by asterisks (* = P < 0.05; ** = P < 0.01; *** = P < 0.001).


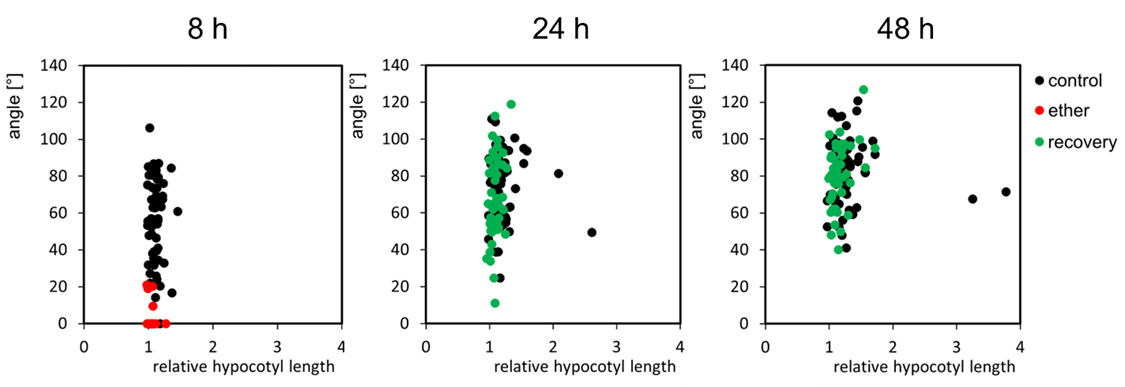


**Supplementary Fig. S4 Graphs of the dependence of phototropic bending on relative hypocotyl elongation in control, etherised, and recovered 5-day-old** ***A. thaliana* seedlings at 8 h, 24 h, and 48 h.** The data shown come from all experiments, i.e., with etiolated and de-etiolated seedlings. Correlation coefficient of phototropic angle and hypocotyl elongation for all measured seedlings together: r = 0.24 (n = 330).


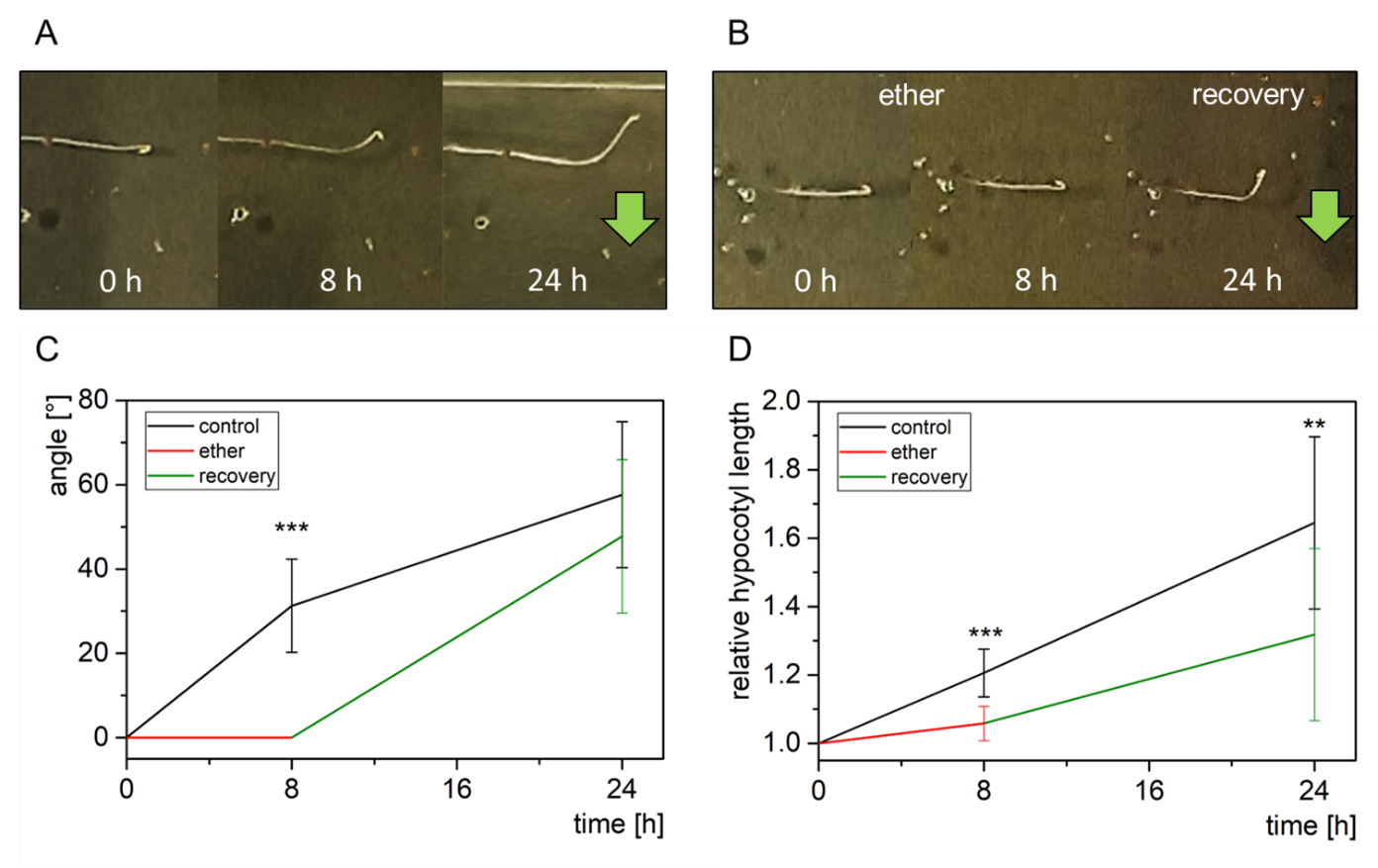


**Supplementary Fig. S5 Gravitropic response of 5-day-old *A. thaliana* etiolated hypocotyls under diethyl ether anaesthesia.** Gravitropic response of control (A) and etherised (B) seedling (after 8 h removed from diethyl ether to recover). Representative images are shown. The green arrows depict the direction of gravity vector. (C) Gravitropic bending and (D) relative hypocotyl length of control (black lines), etherised (red lines), and recovered (green lines) seedlings. Data depict means ± SD from a single representative experiment (n = 10–20). Significant differences between control and etherised seedlings evaluated by Student t-test are denoted by asterisks (* = P < 0.05; ** = P < 0.01; *** = P < 0.001).

**
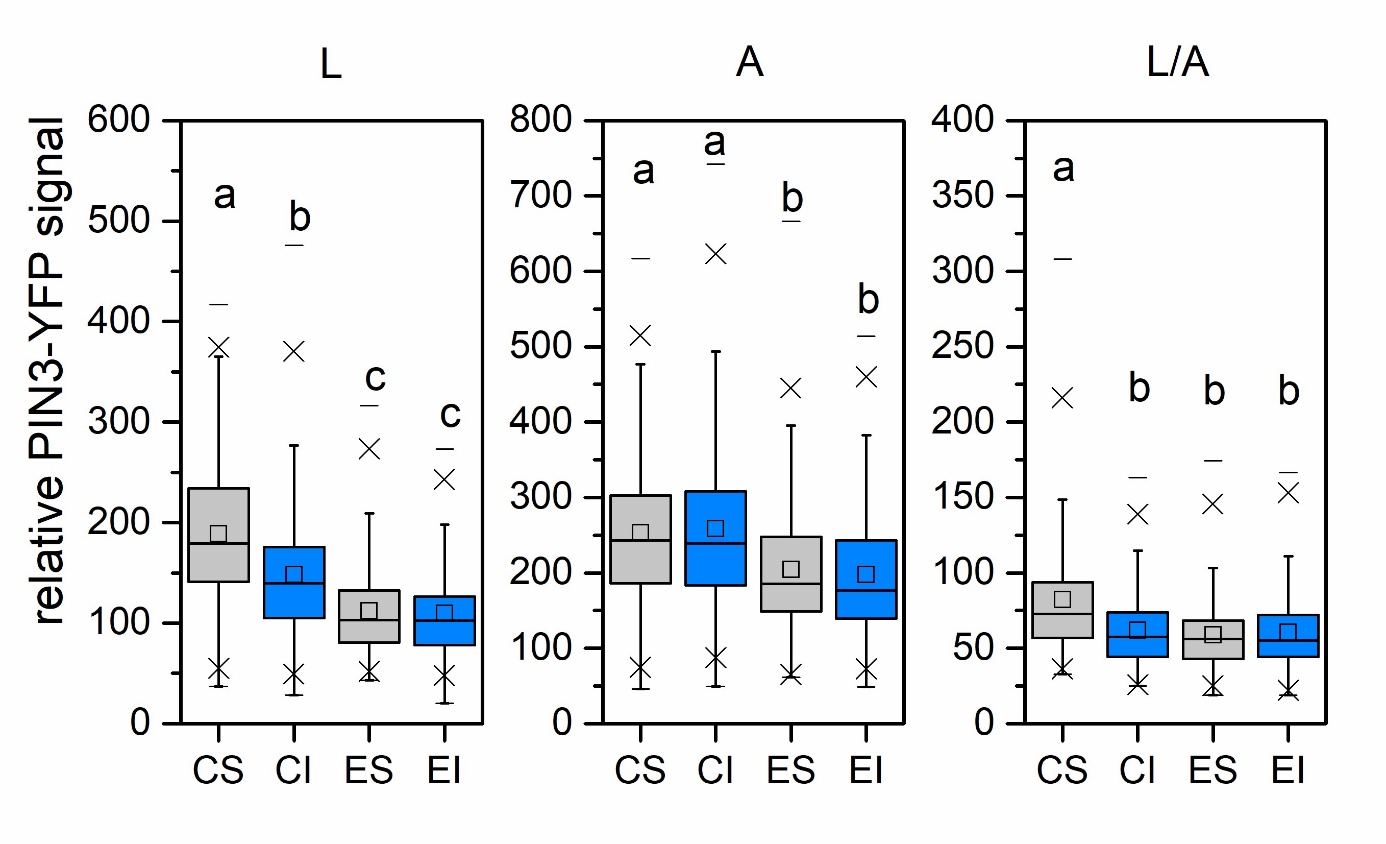
**

**Supplementary Fig. S6 PIN3-YFP signal in response to unilateral LBL (1 μmol m^-2^ s^-1^) in upper hypocotyl endodermal membranes of 5-day-old transgenic *PIN3::PIN3-YFP A. thaliana* seedlings.** (CS) control shaded cells, (CI) control illuminated cells, (ES) etherised shaded cells, (EI) etherised illuminated cells. L value: units defined as ${{FI}_{lateral}}/{{FI}_{cytosol}}\times100$, A value: units defined as ${{FI}_{apical}}/{{FI}_{cytosol}}\times100$, L/A ratio: units defined as ${{FI}_{lateral}}/{{FI}_{apical}}\times100$. Box plots represent signals from all analysed seedlings from all measured cells combined (n = 230–350). The squares represent means; the lines represent medians; the whiskers represent range within 1.5 IQR; the x represents first and last percentile; – represents min and max. Significant differences at *P* < 0.05 are indicated using different letters (ANOVA + Tukey test).





**Supplementary Fig. S7 LTI6B-GFP signal in response to unilateral LBL (1 μmol m^-2^ s^-1^) in upper hypocotyl endodermal membranes of 5-day-old** **transgenic *LTI6B::LTI6B-GFP A. thaliana* seedlings.** (CS) control shaded cells, (CI) control illuminated cells, (ES) etherised shaded cells, (EI) etherised illuminated cells. L value: units defined as ${{FI}_{lateral}}/{{FI}_{cytosol}}\times100$, A value: units defined as ${{FI}_{apical}}/{{FI}_{cytosol}}\times100$, L/A ratio: units defined as ${{FI}_{lateral}}/{{FI}_{apical}}\times100$. Box plots represent signals from all analysed seedlings from all measured cells combined (n = 110–125). The squares represent means; the lines represent medians; the whiskers represent range within 1.5 IQR; the x represents first and last percentile; – represents min and max. Significant differences at *P* < 0.05 are indicated using different letters (ANOVA + Tukey test).
